# Rule for Examination of Urine

**Published:** 1883-07

**Authors:** 


### Rule for Examination of Urine.

1. Sediment in the urine has no significance unless deposited within twenty-four hours.

2. Albumen in the urine does not indicate kidney disease unless accompanied by tube-casts. The most fatal form of Bright's disease—contracted kidney—has little or no albumen.

3. Every white crystal in urine, regardless of shape, is a phosphate, except the oxalate of lime, which has its own peculiar form, urine alkaline.

4. Every yellow crystal is uric acid if the urine is acid, or a urate if the urine is alkaline.

5. Mucous casts, pus, and epithelium signify disease of the bladder (cystitis) or of other parts of the urinary tract, as determined by the variety of epithelium.

6. The urine from females can often be differentiated from the urine of the male, by finding in it the tesselated epithelium of the vagina.

7. Hyaline casts (narrow), blood, and epithelial casts signify acute catarrhal nephritis. Much albumen.

8. Broad hyaline casts and epithelial dark, granular and oil casts signify chronic catarrhal nephritis. At first, much albumen; later less.

9. Hyaline and pale granular casts and little or no albumen signify interstitial nephritis.

10. Broader casts are worse than narrow casts, as far as diagnosis is concerned, for the former signify a chronic disease.

11. The urine should be fresh for microscopical examination, as the micrococci will change hyaline casts into granular casts or devour them entirely in a short time.

12. Uric acid in the urine may, in Trommer's test for sugar, form a protoxide of copper, thus often deceiving the examiner in the belief that he has discovered sugar. Thus when urine shows only a trace of sugar, other methods of examinations besides the Trommer's must be used—preferably the lead test.

13. The microscope gives us better ideas of the exact condition of affairs in the examination of urine than the various chemical tests. Therefore the time has come when every true physician should know how to handle a microscope.—*Dr. Formad, Louisville Med. News.*

